# Plasma 25-hydroxyvitamin D and risk of premenstrual syndrome in a prospective cohort study

**DOI:** 10.1186/1472-6874-14-56

**Published:** 2014-04-12

**Authors:** Elizabeth R Bertone-Johnson, Susan E Hankinson, Nancy G Forger, Sally I Powers, Walter C Willett, Susan R Johnson, JoAnn E Manson

**Affiliations:** 1 Division of Biostatistics and Epidemiology, Department of Public Health, School of Public Health and Health Sciences, University of Massachusetts, 409 Arnold House, 715 North Pleasant Street, Amherst, MA 01003, USA; 2 Channing Division of Network Medicine, Department of Medicine, Brigham and Women’s Hospital and Harvard Medical School, Boston, MA 02115, USA; 3 Department of Epidemiology, Harvard School of Public Health, Boston, MA 02115, USA; 4 Neuroscience Institute, Georgia State University, Atlanta, GA 30302, USA; 5 Department of Psychology, University of Massachusetts, Amherst, MA 01003, USA; 6 Department of Nutrition, Harvard School of Public Health, Boston, MA 02115, USA; 7 Department of Obstetrics and Gynecology, University of Iowa, Iowa City, IO 52242, USA; 8 Division of Preventive Medicine, Department of Medicine, Brigham and Women’s Hospital and Harvard Medical School, Boston, MA 02115, USA

**Keywords:** Vitamin D, Calcium, Premenstrual syndrome, Prospective studies

## Abstract

**Background:**

Moderate to severe premenstrual syndrome (PMS) affects 8–20 percent of premenopausal women. Previous studies suggest that high dietary vitamin D intake may reduce risk. However, vitamin D status is influenced by both dietary vitamin D intake and sunlight exposure and the association of vitamin D status with PMS remains unclear.

**Methods:**

We assessed the relation of plasma 25-hydroxyvitamin D (25OHD), total calcium and parathyroid hormone levels with risk of PMS and specific menstrual symptoms in a case–control study nested within the prospective Nurses’ Health Study II. Cases were 401 women free from PMS at baseline who developed PMS during follow-up (1991–2005). Controls were women not experiencing PMS (1991–2005), matched 1:1 with cases on age and other factors. Timed luteal phase blood samples were collected between 1996 and 1999 from cases and controls. We used conditional logistic regression to model the relation of 25OHD levels with risk of PMS and individual menstrual symptoms.

**Results:**

In analyses of all cases and controls, 25OHD levels were not associated with risk of PMS. However, results differed when the timing of blood collection vs. PMS diagnosis was considered. Among cases who had already been diagnosed with PMS at the time of blood collection (n = 279), 25OHD levels were *positively* associated with PMS, with each 10 nmol/L change in 25OHD associated with a 13% higher risk. Among cases who developed PMS after blood collection (n = 123), 25OHD levels were unrelated to risk of PMS overall, but inversely related to risk of specific menstrual symptoms. For example, each 10 nmol/L increase was associated with a significant 21% lower risk of breast tenderness (P = 0.02). Total calcium or parathyroid hormone levels were unrelated to PMS.

**Conclusions:**

25OHD levels were not associated with overall risk of PMS. The positive association observed among women already experiencing PMS at the time of 25OHD measurement is likely due to confounding by indication related to use of dietary supplements to treat menstrual symptoms. Results from prospective analyses, which were less likely influenced by this bias, suggest that higher 25OHD levels may be inversely related to the development of specific menstrual symptoms.

## Background

Approximately 8-20% percent of premenopausal women experience premenstrual syndrome (PMS), [1,2] a disorder of moderate to severe symptoms in the luteal phase of the menstrual cycle that substantially interfere with normal life activities and interpersonal relationships [3]. Symptoms are diverse, and commonly include affective symptoms such as irritability, mood swings, anxiety and depression; physical symptoms such as breast tenderness, bloating, and headaches; and behavioral symptoms such as insomnia, changes in appetite, and difficulty concentrating. While pharmacologic treatments including anti-depressants and oral contraceptives are frequently used to treat PMS, these have substantial side effects and none has a reported efficacy greater than 60-70% [2,3]. Because of these limitations, effective non-pharmaceutical strategies for preventing and treating PMS are needed.

Previous studies have evaluated the role of vitamin D in preventing and/or treating mood and gynecologic disorders that share common features with PMS. Multiple studies have reported inverse associations between vitamin D status and risk of depression, [4,5] fibromyalgia, [6] dysmenorrhea, [7] and uterine fibroids [8,9]. However, it remains unknown whether vitamin D may be useful for preventing or treating PMS. Our research group previously reported that women who consumed approximately 400 IU of vitamin D per day had a significant 40% lower risk of being diagnosed with PMS in the next 2–4 years, as compared to women consuming approximately 100 IU/day [10]. This association was present even after adjustment for calcium, which has been shown to beneficially impact PMS symptoms in some [11-14] but not all studies [15]. While these results suggest that vitamin D may be related to the development of PMS, many questions remain and must be addressed before vitamin D can be recommended for PMS. Vitamin D status is the product of cutaneous vitamin D production and not just dietary intake. Correlations of dietary vitamin D intake with the main circulating vitamin D metabolite, 25-hydroxyvitamin D (25OHD), are relatively low, [16] indicating that many factors influence vitamin D metabolism and availability [17,18]. Thus, assessing dietary vitamin D intake alone may be insufficient to accurately characterize a woman’s vitamin D status.

Few studies have evaluated how 25OHD levels are associated with PMS or with premenstrual dysphoric disorder (PMDD), and results have been inconsistent [19-21]. Importantly, because previous studies have been limited to women already experiencing PMS at the time of their 25OHD measurement, results from these studies may be confounded by patients’ use of vitamin D or multivitamin supplements or other dietary changes to treat PMS symptoms, as is currently recommended [22,23]. Prospective studies evaluating 25OHD levels in women prior to PMS development are needed to better understand whether vitamin D may be etiologically related to the development of PMS.

We have evaluated whether plasma levels of 25OHD, along with two vitamin D-related biomarkers (total calcium and parathyroid hormone), are associated with PMS among a subset of participants in the Nurses’ Health Study II (NHS2). We have assessed relations overall, and after taking the timing of 25OHD measurement vs. PMS diagnosis into consideration. Finally, because the physical, affective, and behavioral symptoms of PMS likely have different etiologies, we have assessed whether 25OHD levels are differently associated with specific symptoms of PMS.

## Methods

### Study population

The NHS2 is an ongoing prospective cohort study of 116,686 US female registered nurses who responded to a mailed questionnaire in 1989 when they were 25–42 years old. On the baseline questionnaire, participants provided information on their medical history and health-related behaviors including smoking and oral contraceptive use. Cohort members have completed questionnaires every two years thereafter to update information on risk factors and to identify new diagnoses of disease. All research protocols for the NHS2, under which data for the present study were collected, were approved by the Institutional Review Board at Brigham and Women’s Hospital, Boston, MA. Additionally, protocols specific to the data analyses included in this project were approved by Institutional Review Boards at Brigham and Women’s Hospital and the University of Massachusetts. The NHS2 is not a publically-available database, but access to raw data is considered for sharing under established protocols in place at the Channing Laboratory, Brigham and Women’s Hospital in compliance with the NIH Grants Policy on Sharing Unique Research Resources.

### Assessment of premenstrual syndrome

**Figure 1:**
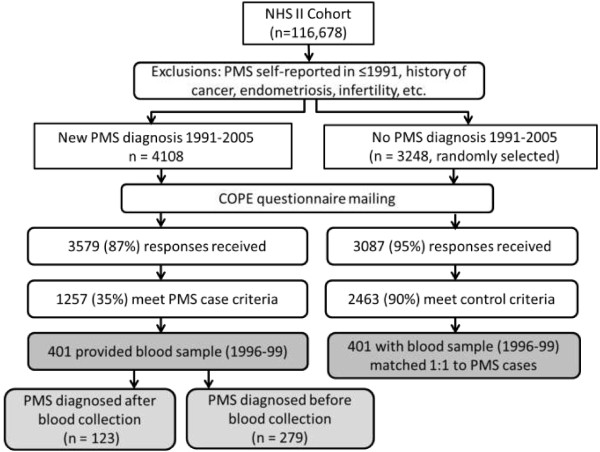
Selection of Nurses’ Health Study II participants into the premenstrual syndrome Sub-study and analysis of 25-hydroxyvitamin D level and risk of PMS.

Women included in this analysis participated in the NHS2 PMS Sub-Study, described in detail previously [10,24]. Briefly, we identified all NHS2 members who had not reported a diagnosis of PMS on their main NHS2 questionnaire in either 1989 or 1991, and were thus at risk for being diagnosed with PMS during follow-up. We then identified all premenopausal women who reported on a NHS2 questionnaire between 1991 and 2005 that they had been newly diagnosed with PMS by a physician (n = 4,108; Figure 1). As a comparison group, we randomly selected 3,248 premenopausal women who did not report PMS during the follow-up period (1991–2005). To reduce the likelihood of including women with PMS-type symptoms caused by conditions other than PMS, from both groups we excluded women who self-reported: a diagnosis of cancer other than non-melanoma skin cancer; hysterectomy; infertility; endometriosis; or menstrual cycles that were too irregular to determine length.

Participants were sent a questionnaire based on the Calendar of Premenstrual Experiences, [25] on which we assessed the occurrence of 26 physical, behavioral and affective symptoms, symptom timing during the menstrual cycle, and the impact of symptoms on multiple domains of daily functioning. Completed questionnaires were received from 3,579 (87%) of the women self-reporting PMS and 3,087 (95%) women in the comparison group. We used responses to further limit our case group to women who met established criteria for moderate to severe PMS [25]. Case criteria included: 1) the occurrence of at least one physical/behavioral and one affective menstrual symptom; 2) overall symptom severity of moderate or severe, or symptom impact on one or more life activities and social relationships rated as moderate or severe; 3) symptoms beginning within 14 days before onset of menses; 4) symptoms ending within 4 days after onset of menses; and 5) symptoms absent in the week after menses ends. Overall, 1,257 (35%) self-reported cases met these additional criteria (Figure 1). The proportion of self-reported PMS cases meeting our validation criteria is consistent with those of other recent population-based studies of PMS and calcium/vitamin D [12,19].

We also used menstrual symptom questionnaires to further limit the comparison group to women who confirmed that they experienced no menstrual symptoms or only mild symptoms of no personal impact. Ultimately, 2,463 (80%) comparison women met these control criteria. Women who did not meet either case or control criteria were excluded from further analysis. This procedure allowed us to compare women at extreme ends of the spectrum of menstrual symptom experience, thereby minimizing the likelihood of misclassification between cases and controls.

The validity of our approach to identifying PMS cases and controls was assessed previously among 135 sub-study members first reporting PMS in 2001 and 371 not reporting PMS (1989–2001) [24]. Cases meeting our criteria for PMS were very similar to cases also reporting clinician-supervised prospective symptom charting, in terms of symptom frequency (e.g., mean number of physical symptoms: 5.5 vs. 6.1, P > 0.05), timing of occurrence (e.g., mean number of days symptoms began before onset of menses: 5.7 vs. 6.1, P > 0.05), and severity (e.g., symptoms caused moderate-severe social isolation: 10% vs. 17%, P > 0.05). Furthermore, odds ratios for the associations of age and calcium intake with PMS risk using both case definitions were nearly identical, suggesting that our method is comparable to prospective charting in its ability to classify PMS cases and controls in large epidemiologic studies.

### Blood sample collection

From 1996–1999, all members of the NHS2 cohort who had not been diagnosed with cancer were invited to provide blood samples [26]. Women who were premenopausal, not using oral contraceptives or other hormones, and who had not been pregnant or breast feeding in the past 6 months were asked to collect two timed blood samples from a single menstrual cycle. The luteal phase sample, used in the present analysis, was collected 7–9 days before the anticipated start of the next menstrual period. We sent women the supplies needed to collect and return samples via overnight courier, including heparinized tubes and an ice pack to keep samples cool. Upon receipt, samples were centrifuged, separated into blood components, and archived at -130°C or colder. Samples were ultimately received from 29,611 NHS2 members. Participants providing a blood sample did not differ from the main NHS2 cohort in terms of body mass index (26 vs. 26 kg/m^2^), ever smoking (34% vs. 36%), history of oral contraceptive use (86% vs. 88%), parity (1.9 vs. 1.9 children) and other factors [26].

Of the 1,257 PMS cases included in the PMS Sub-study, 401 provided blood samples (Figure 1). Cases providing blood samples did not differ from those not providing blood samples (n = 856) in terms of age in 1999 (42 vs. 42 years), body mass index (26.0 vs. 26.6 kg/m^2^), ever smoking (42% vs. 38%), vitamin D intake (378 vs. 380 IU/day) and other factors (all P > 0.05). Blood samples were collected before PMS diagnosis for 123 cases (median duration between blood collection and later diagnosis = 28 months). For the remaining 279 cases, blood was collected after PMS diagnosis (median duration between diagnosis and later blood collection = 22 months). We then matched cases 1:1 with controls from the PMS Sub-Study by age, month of blood collection and follow-up time. If more than one control met matching criteria for a single case, control selection among eligible matches was made randomly.

### Laboratory measurement

Plasma samples were analyzed for 25OHD, total calcium and intact parathyroid hormone (iPTH) levels in the laboratory of Dr. Nader Rifai (Children’s Hospital, Boston, MA). 25OHD was measured by an enzyme immunoassay from Immunodiagnostic Systems Inc. (Fountain Hills, AZ). This assay is primarily a measure of the D_3_ isoform of 25OHD, but is also cross-reactive for the D_2_ isoform. Cross-reactivity for the two isoforms are 100% (D_3_) and 75% (D_2_). Total calcium was measured by a colorimetric assay on the Hitachi 917 analyzer using Roche reagents (Roche Diagnostics, Indianapolis, IN). Intact PTH was measured by an electrochemiluminescence immunoassay on the 2010 Elecsys autoanalyzer (Roche Diagnostics, Indianapolis, IN). Case and control pairs were blinded, assayed together, randomly ordered within boxes. We also included blinded split samples from a plasma pool to assess quality control. Coefficients of variation were 8% for 25OHD, 5% for total calcium, and 14% for iPTH.

### Assessment of other factors

Information on a variety of demographic, reproductive and lifestyle factors has been collected regularly from NHS2 members. Factors measured at baseline included age, race/ethnicity, skin type/pigmentation, height, and age at menarche. Information on full term pregnancies, age at first birth, oral contraceptive use, and smoking history was collected at baseline and updated every two years. Participants reported their current weight on each biennial questionnaire, which we used to calculate body mass index (BMI; weight in kg/height in meters squared). Participation in physical activity was measured in 1991, 1997 and 2001 by asking how much time women spent each week participating in specific recreational activities. Responses were used to calculate metabolic equivalent task (MET) hours of activity [27].

Use of medications, including anti-depressants, was assessed from 1993 onward. History of clinician-diagnosed depression was reported on main NHS2 questionnaires in 2003 and 2005, as well as on the menstrual symptom questionnaire. Socioeconomic factors including mother’s and father’s education level were reported in 2005. Food and nutrient intake was assessed every four years by validated semi-quantitative foods frequency questionnaire, [28] and used to estimate intake of calcium, vitamin D, potassium, B vitamins and other nutrients from foods and supplements. In 1999, dose categories for calcium on the food frequency questionnaire were none, <400 mg/day, 400-900 mg/day, 901-1300 mg/day, and ≥1301 mg/day. Use of vitamin D supplements was measured as yes/no. All nutrients were adjusted for total energy intake using the residual method [29].

### Statistical analysis

All statistical analyses were conducted with SAS (SAS Institute, Inc., Cary, North Carolina). We first compared characteristics at the time of blood collection of all PMS cases vs. controls using t-tests. We characterized vitamin D status in several ways. First, to assess the impact of vitamin D deficiency vs. sufficiency on risk, we divided participants into groups based on current Institute of Medicine guidelines (<50 vs. ≥ 50 nmol/L) [30]. To evaluate the impact of more extreme levels of 25OHD on risk, we divided participants into quintiles based on the distribution in the control group. Finally, we assessed the linear relation of 25OHD with risk of PMS by modeling 25OHD as a continuous variable. In each analysis, we estimated odds ratios (OR) for PMS by 25OHD level using conditional logistic regression, and calculated 95% confidence intervals (CI). P values <0.05 were considered statistically significant.

We used multivariable conditional logistic regression to control for the effects of other factors that could confound the vitamin D-PMS relationship, or that have been associated previously with PMS in our population. For factors that could change during follow-up (e.g., physical activity, BMI, smoking status), we modeled level at the time of blood collection. In addition to matching factors (age, month of blood collection and follow-up time), our final model included race/ethnicity, geographic region of residence, number of moles on lower leg (a proxy measure of skin tone, sun sensitivity, and potentially of early life sun exposure [31,32]), BMI, physical activity, alcohol intake, current and former smoking, history of oral contraceptive use, maternal education, antidepressant use, childhood trauma, and vitamin B6 intake. We repeated these analyses evaluating the association of total calcium and iPTH levels with risk of PMS, and adjusted each biomarker for the effects of the others.

The association of biomarkers and PMS could be influenced by many issues relating to timing of blood collection vs. PMS diagnosis, including use of calcium and vitamin D supplements to treat existing PMS. We thus repeated our main analyses separately for cases who had already been diagnosed with PMS by the time of blood collection and cases with blood collected before their PMS diagnosis. To maximize power for these comparisons, we compared each case group to all controls using unconditional logistic regression, and further adjusted for age, season of blood collection, and follow-up time in our multivariable models.

Finally, to determine if 25OHD levels were differently related to individual symptoms of PMS, we assessed risk of each symptom queried. In these analyses, we compared cases experiencing each symptom to controls not experiencing the symptom in multivariable unconditional logistic regression models. Finally, we conducted a sensitivity analysis excluding women reporting any clinical diagnosis of depression during follow-up (n = 173 cases and 63 controls excluded), to determine whether associations were robust to potential misclassification of depression as PMS.

## Results

Characteristics of PMS cases and controls measured at the time of blood collection are presented in Table 1. Overall, cases were more likely than controls to be current or former smokers (P < 0.001 for both). Cases had a marginally higher mean daily alcohol intake (4.6 vs. 3.7 g/day; P = 0.05), and were more likely to report significant trauma in childhood (18.1% vs. 5.6%; P < 0.0001). Though the study population was racially homogenous overall, cases and controls differed significantly in the proportion of participants reporting white race (P = 0.02). Both anti-depressant use and oral contraceptive use were more commonly reported by cases than controls (P < 0.001 for both), as was use of B vitamin supplements (P = 0.002). Cases and controls did not differ by age, BMI, age at menarche, or physical activity. Mean levels of vitamin D-related biomarkers did not differ between cases and controls.

**Table 1:** Characteristics of premenstrual syndrome cases and controls at the time of blood collection, NHS2 PMS Sub-study

**Characteristic**	**PMS cases n = 401**	**Controls n = 401**	**P value**
	Mean (SD)	Mean (SD)	
Age (years)	40.6 (4.0)	40.7 (3.8)	0.94
Body mass index (kg/m^2^)	25.7 (5.8)	25.4 (5.8)	0.50
Body mass index at age 18 (kg/m^2^)	21.5 (3.4)	21.4 (3.5)	0.81
Age at menarche (years)	12.5 (1.4)	12.4 (1.5)	0.45
Number of full term pregnancies	2.0 (1.2)	2.0 (1.3)	0.43
Age at first birth (years)*	26.2 (4.2)	26.7 (4.1)	0.11
Physical activity (MET/wk)	18.7 (19.4)	20.0 (20.8)	0.36
Alcohol intake (grams/day)	4.6 (6.8)	3.7 (6.5)	0.05
	%	%	
White race	96.0	98.8	0.02
Mother had > high school education	31.9	39.9	0.02
More than 5 moles on lower leg†	15.5	17.0	0.57
Low UV radiation index region of US	22.2	26.0	0.22
Current smoker	11.2	4.7	<0.001
Former smoker	30.7	15.7	<0.001
Tubal ligation	31.1	25.2	0.06
Significant childhood trauma	18.1	5.6	<0.001
Antidepressant use&	14.7	6.2	<0.001
Ever used oral contraceptives	86.5	77.3	<0.001
Calcium/vitamin D supplement use	41.4	39.9	0.67
B vitamin supplement use	17.7	10.0	0.002
Regular multivitamin use	48.9	44.1	0.18
**Biomarkers**	Mean (SD)	Mean (SD)	
25-hydroxyvitamin D (nmol/L)	66.5 (20.5)	64.8 (19.9)	0.24
Total calcium (mg/dL)	9.74 (0.39)	9.72 (0.38)	0.64
Intact Parathyroid hormone (pg/mL)	31.2 (11.5)	31.1 (11.6)	0.92

*Among parous women (n = 326 controls, 340 cases).

†At baseline; a measure of light skin tone.

&Use before diagnosis (cases).

In our main analyses of all PMS cases and their matched controls, we did not find plasma levels of 25OHD, total calcium or parathyroid hormone to be associated with PMS (Table 2). In unadjusted analyses, the OR for women in the highest quintile of 25OHD (median = 91.6 nmol/L) was 1.26 (95% CI = 0.79 – 2.02) compared to the lowest quintile (median = 42.4 nmol/L). Adjustment for other factors including BMI, smoking, and physical activity did not materially alter results. For example, after adjustment, women in the highest quintile of 25OHD had an OR of 1.16 (95% CI = 0.60 – 2.41). Results for 25OHD classified into broader categories indicating vitamin D deficiency vs. sufficiency (i.e., <50 vs. ≥50 nmol/L) were similarly null. Neither total calcium nor iPTH levels were associated with risk of PMS.

**Table 2:** Odds ratios (OR) for premenstrual syndrome by blood levels of vitamin D-related biomarkers, NHS2 PMS Sub-study

**Factor**	**Cases**	**Controls**	**Unadjusted OR (95% CI)**	MV adjusted OR* (95% CI)
**25-hydroxyvitamin D**				
Deficiency vs. Sufficiency				
< 50 nmol/L	86	90	1.00	1.00
≥ 50 nmol/L	315	311	1.07 (0.75 – 1.53)	1.14 (0.77 – 1.68)
Quintiles (median, nmol/L)				
Q1 (42.4)	72	81	1.00	1.00
Q2 (53.5)	76	79	1.10 (0.70 – 1.74)	1.02 (0.56 – 1.84)
Q3 (62.6)	72	81	1.03 (0.65 – 1.63)	1.23 (0.67 – 2.29)
Q4 (73.6)	96	79	1.44 (0.90 – 2.30)	1.25 (0.65 – 2.41)
Q5 (91.6)	85	81	1.26 (0.79 – 2.02)	1.16 (0.60 – 2.41)
Per 10 nmol/L change	401	401	1.05 (0.97 – 1.13)	1.04 (0.93 – 2.25)
**Total calcium**				
Quintiles (median, mg/dL)				
Q1 (9.3)	79	79	1.00	1.00
Q2 (9.6)	83	83	1.00 (0.65 – 1.55)	1.13 (0.63 – 2.02)
Q3 (9.7)	90	101	0.91 (0.59 – 1.39)	0.91 (0.52 – 1.60)
Q4 (9.9)	67	63	1.09 (0.67 – 1.77)	1.28 (0.69 – 2.37)
Q5 (10.2)	82	75	1.12 (0.69 – 1.80)	1.03 (0.55 – 1.94)
Per 1 mg/dL change	401	401	1.11 (0.74 – 1.67)	1.18 (0.70 – 2.00)
**iPTH**				
Quintiles (median, pg/mL)				
Q1 (19.4)	81	80	1.00	1.00
Q2 (23.8)	59	79	0.72 (0.46 – 1.15)	0.70 (0.38 – 1.27)
Q3 (28.5)	96	79	1.21 (0.79 – 1.85)	1.50 (0.85 – 2.64)
Q4 (34.5)	80	80	1.00 (0.64 – 1.55)	1.16 (0.65 – 2.08)
Q5 (46.9)	85	83	1.01 (0.66 – 1.55)	0.89 (0.50 – 1.58)
Per 10 pg/mL change	401	401	1.01 (0.89 – 1.13)	0.93 (0.78 – 1.10)

*Multivariable (MV) OR from conditional logistic regression adjusted for race/ethnicity, geographic region, BMI, physical activity, alcohol intake, smoking status, number of moles on leg, oral contraceptive use, maternal education, antidepressant use, significant childhood trauma, and vitamin B6 intake. Each biomarker adjusted for the others (continuous levels).

We then evaluated associations separately by timing of 25OHD measurement vs. PMS diagnosis (Table 3). 25OHD levels were higher in cases who had already been diagnosed with PMS as the time of their blood collection (67.9 nmol/L) as compared to controls (64.8 nmol/L), and 25OHD was positively associated with PMS risk. Specifically, each 10 nmol/L increase in 25OHD was associated with a 13% higher risk of PMS (95% CI = 1.03 – 1.25). In contrast, 25OHD levels were not associated with risk in cases with blood collected before diagnosis.

**Table 3:** Odds ratios for premenstrual syndrome and individual menstrual symptoms, stratified by timing of 25OHD measurement vs. PMS diagnosis

	**25OHD measured before PMS diagnosis**		**25OHD measured after PMS diagnosis**	
**Outcome**	Cases*	**MV OR† (95% CI) for 10 nmol/L change in 25OHD**	Cases*	**MV OR† (95% CI) for 10 nmol/L change in 25OHD**
Premenstrual Syndrome	123	0.97 (0.85 – 1.11)	279	**1.13 (1.03 – 1.25)**
*Physical & behavioral menstrual symptoms*				
Swelling of extremities	27	0.77 (0.57 – 1.06)	58	1.12 (0.96 – 1.31)
Breast tenderness	79	**0.79 (0.65 – 0.96)**	211	1.05 (0.93 – 1.19)
Fatigue	64	**0.80 (0.66 – 0.97)**	153	1.10 (0.99 – 1.23)
Diarrhea/constipation	50	**0.80 (0.65 – 0.98)**	108	**1.13 (1.00 – 1.29)**
Backache	41	0.85 (0.67 – 1.07)	104	1.11 (0.98 – 1.26)
Bloating	77	0.85 (0.71 – 1.03)	181	1.10 (0.99 – 1.23)
Hot flashes	16	0.86 (0.59 – 1.25)	15	1.09 (0.79 – 1.52)
Palpitations	15	0.90 (0.62 – 1.32)	22	1.14 (0.90 – 1.45)
Food cravings	82	0.92 (0.78 – 1.09)	204	**1.15 (1.03 – 1.29)**
Appetite changes	68	0.93 (0.79 – 1.10)	163	**1.17 (1.04 – 1.31)**
Forgetfulness	24	0.96 (0.72 – 1.28)	58	1.15 (0.99 – 1.34)
Insomnia	33	0.95 (0.76 – 1.20)	47	1.15 (0.97 – 1.36)
Acne	36	0.97 (0.76 – 1.24)	96	**1.14 (1.01 – 1.29)**
Headache	58	1.02 (0.85 – 1.23)	125	1.07 (0.95 – 1.22)
*Affective menstrual symptoms*				
Depression	51	**0.81 (0.66 – 1.00)**	117	1.10 (0.98 – 1.24)
Anxiety	35	0.82 (0.63 – 1.06)	91	**1.19 (1.04 – 1.35)**
Tendency to cry easily	59	0.92 (0.76 – 1.12)	158	**1.13 (1.01 – 1.27)**
Anger	68	0.94 (0.78 – 1.13)	165	**1.12 (1.01 – 1.25)**
Irritability	110	0.95 (0.81 – 1.11)	235	**1.19 (1.07 – 1.34)**
Hypersensitivity	37	0.95 (0.76 – 1.19)	112	**1.14 (1.01 – 1.29)**
Cramping	46	0.98 (0.80 – 1.20)	92	1.03 (0.90 – 1.19)
Mood swings	76	1.00 (0.85 – 1.17)	191	**1.15 (1.04 – 1.28)**
Desire for aloneness	38	1.01 (0.80 – 1.28)	113	**1.14 (1.00 – 1.29)**

Odds ratios (OR) correspond to change in risk of PMS or individual symptoms for each 10 nmol/L change in 25OHD level. OR significant at P < 0.05 are bolded.

*Number of PMS cases reporting specific symptom. Results for symptoms of dizziness, nausea, confusion are not shown, as these symptoms were reported by ≤10 cases.

†Multivariable (MV) OR from unconditional logistic regression adjusted for age, season of blood collection, race/ethnicity, geographic region, BMI at blood draw, physical activity, alcohol intake, smoking status at blood collection, number of moles on leg, oral contraceptive use, maternal education, antidepressant use, significant childhood trauma, and vitamin B6 intake, and plasma levels of total calcium and parathyroid hormone.

Relations of 25OHD levels with individual menstrual symptoms also varied by timing of blood collection. Among women with 25OHD measured before they were diagnosed with PMS, plasma 25OHD levels were inversely related to risk of developing breast tenderness, fatigue, diarrhea and/or constipation, and depression (all P < 0.05; Table 3). For example, each 10 nmol/L increase in 25OHD level was associated with a significant 21% lower risk of developing breast tenderness (P = 0.02), and a significant 19% lower risk of depression (P = 0.047). In contrast, among women who already had PMS at the time of blood collection, 25OHD was positively associated with risk of diarrhea/constipation, food cravings, appetite changes, and a variety of affective symptoms (all P < 0.05). For example, each 10 nmol/L increase in 25OHD level was associated with a significant 19% higher risk of anxiety and irritability (P < 0.01 for both).

For all biomarkers, results from sensitivity analyses excluding women reporting a diagnosis of depression during follow-up were similar to the main analyses. For example, the OR for each 10 nmol/L change in 25OHD was 1.08 (95% CI = 0.98 – 1.19).

## Discussion

Overall, we found no association between plasma levels of 25OHD and risk of PMS in our longitudinal study. However, results differed somewhat based on the timing of blood measurement in relation to PMS diagnosis. Among women who had already been diagnosed with PMS by the time of blood collection, 25OHD levels were positively associated with PMS risk. In contrast, among women diagnosed with PMS after blood draw, 25OHD levels were not associated with future risk of developing PMS.

The observed positive association of 25OHD with PMS risk was contrary to our hypothesis, and reasons for this finding are unclear. One potential explanation is that women experiencing PMS may treat their symptoms with calcium and vitamin D supplements, or multivitamins containing these nutrients, as is currently recommended by the American Congress of Obstetricians and Gynecologists and other groups [22,23]. Consequently, 25OHD levels would elevated in PMS cases due to differences in supplementation practices, and this type of confounding by indication would bias results. In our study, mean 25OHD levels were indeed significantly higher in women who had PMS at blood collection (67.9 nmol/L) compared to those not yet diagnosed (63.2 nmol/L; P = 0.04), suggesting that supplementation, diet and/or behaviors influencing vitamin D status differed between case groups.

We further explored this hypothesis in *post hoc* analyses. At the time of blood collection, calcium and vitamin D supplement use was reported by 43.5% of women already experiencing PMS, compared to 36.6% of women not yet diagnosed (P = 0.19). Multivitamin use also varied slightly between groups (50.% vs. 44.7%, respectively; P = 0.27). This finding is consistent with results from Thys-Jacobs *et al.*[20], who reported non-significantly higher use of calcium supplements (46% vs. 32%) and vitamin D supplements (41% vs. 30%) among women with PMDD compared to symptom-free controls [20]. Among calcium supplement users in our population, 56% reported taking 400–900 mg/day, while 36% reported taking >900 mg/day; this level of supplementation may have considerable impact on associations.

When we took supplementation practices into consideration among cases already diagnosed with PMS at the time of blood collection, we found the positive association to be limited to women using supplements (OR for each 10 nmol/L change = 1.19; P = 0.002). In contrast, among non-users of supplements, 25OHD levels were unrelated to risk (OR = 1.03; P = 0.67). These results support the hypothesis that women experiencing PMS may be self-medicating with dietary supplements. This interpretation is also consistent with results from a previous study of B vitamin supplementation and PMS in our population, in which we found vitamin B6 supplement use to be more common among women already experiencing PMS than among women not yet diagnosed with PMS [33]. The likelihood of confounding by indication suggests a need for caution in interpreting results from case–control and cross-sectional studies of nutritional factors and PMS. It also underscores the importance of evaluating these relations prospectively whenever possible, to minimize the chance of bias.

Over 100 different menstrual symptoms may occur in PMS, [34] and the specific symptoms experienced by individual women may differ substantially. Because physical, affective, and behavioral symptoms likely have different etiologies, it is may be important to evaluate the associations of etiologic factors with individual symptoms and symptom clusters separately. This hypothesis is supported by randomized trials of pharmacologic therapies for PMS, as reviewed by Halbreich *et al.*[35], which demonstrate substantial variation in treatment response between symptom profiles. For example, selective serotonin reuptake inhibitors appear most beneficial to women experiencing mood symptoms, especially irritability, and behavioral symptoms, but are less effective for treating physical symptoms.

Similarly, associations of vitamin D with specific symptoms may vary. In the present analysis, among women not yet diagnosed with PMS at blood collection, we found 25OHD level to be associated with significantly lower risk of developing breast tenderness, diarrhea/constipation, fatigue and depression (P < 0.05) and possibly with swelling of extremities and bloating (P ≤ 0.11). It is possible that some of these findings may be due to chance, given the relatively large numbers of comparisons we made. Alternatively, differences in observed associations suggest that vitamin D may be associated with some symptoms but not others. For example, clinical studies of PMS suggest that dysfunction of the renin-angiotensin-aldosterone system (RAAS) is involved in premenstrual edema symptoms such as abdominal bloating, swelling of extremities, and breast tenderness [36]. Vitamin D deficiency is also associated with increased RAAS function, contributing to increased fluid balance, blood pressure changes, and hypertension [37,38]. Furthermore, an inverse relation between 25OHD levels and premenstrual depression would be consistent with the literature suggesting that vitamin D may lower risk of unipolar depression [39]. It therefore seems plausible that vitamin D may specifically influence menstrual depression and symptoms related to fluid balance, but not other symptoms. This should be explored in future studies.

Our study has several potential limitations requiring consideration. First, our ability to assess the relation of vitamin D-related biomarkers and PMS may have been limited by access to only a single blood sample collected several years before or after PMS diagnosis. It is possible that the 25OHD level measured in this sample does not reflect long-term vitamin D status. Multiple studies have evaluated the stability of 25OHD within individuals over time [40-43]. For example, in the Nurses’ Health Study, the intraclass correlation comparing three measurements of 25OHD levels collected over 3 years was 0.72 (95% CI = 0.62-0.80) [40]. However, most studies of vitamin D stability in women have been conducted in postmenopausal women or have not directly evaluated variation by menopausal status [40,42,43]. In one study evaluating blood samples drawn before and after menopause in 34 women (median time between samples = 5.8 years) the correlation for 25OHD was 0.39 (P < 0.05), suggesting that levels may be stable over many years, even in the context of substantial changes in levels of estradiol and other sex steroid hormones [41]. While these data suggest that a single 25OHD measurement can accurately represent vitamin D status over multiple years, additional studies specifically evaluating relations in premenopausal women are needed.

Additionally, we were unable to consider the effect of vitamin D binding protein concentrations, which recent studies suggest may importantly affect the bioavailability of 25OHD, and thus relation between vitamin D measurements and health outcomes [44]. Future studies of vitamin D status and PMS risk should incorporate this important parameter. It is important to note that for our prospective analyses, misclassification resulting from the use of a single 25OHD measurement to represent long-term vitamin D status is likely to be non-differential with respect to PMS diagnosis. Consequently, this type of bias would lead to an underestimation of the relation of vitamin D and PMS, as opposed to exaggerating associations. Additional prospective studies, including clinical trials, are needed to clarify the impact of timing of 25OHD measurement on the association of vitamin D and the development of menstrual symptoms and PMS.

Due to the collection and storage protocol used in the NHS2, we were unable to measure ionized calcium, which is a preferred measure of calcium status in clinical populations, and to measure protein stores as they impact calcium status [45]. This may have contributed to some misclassification of the association of calcium status with PMS risk. While we adjusted for a variety of dietary and lifestyle factors that could confound the association of vitamin D and PMS risk, we were unable to separately evaluate indoor and outdoor physical activity (which could differently affect 25OHD levels), or comprehensively assess other aspects of sun exposure. Though we included race/ethnicity, geographic region of residence, and number of moles on lower leg in our multivariable regression models, these factors are likely imprecise measures of sun exposure and some residual confounding may persist.

Our study of PMS is nested within a large, ongoing prospective cohort study of over 116,000 women. Consequently, we were unable to use prospective symptom diaries to classify PMS, as is recommended by the International Society for Premenstrual Disorders [46]. However, we used established criteria to identify women meeting strict criteria for moderate to severe PMS [25] as well as to define a comparison group of women experiencing no menstrual symptoms or few symptoms with no personal impact. Women meeting neither case nor control criteria were excluded from analysis. Thus, the likelihood of misclassification between cases and controls is minimal and is unlikely to bias our findings. Additionally, when we excluded women reporting a diagnosis of depression during follow-up, results were very similar to the main analysis. This suggests that associations were robust to potential misclassification of depression as PMS, or to the impact of comorbid depression on the vitamin D – PMS relation.

## Conclusions

In summary, we did not find 25OHD levels consistent with current IOM guidelines for vitamin D sufficiency to be associated with overall risk of PMS risk. The positive association of 25OHD and risk we observed among women already experiencing PMS at the time of vitamin D measurement is likely due to confounding by indication resulting from the use of dietary supplements to treat menstrual symptoms. Results from prospective analyses, which were likely less influenced by this bias, suggest that higher 25OHD levels may be inversely related to the development of specific menstrual symptoms. Additional prospective studies of vitamin D for the treatment and prevention of PMS are warranted.

## Abbreviations

25OHD: 25-hydroxyvitamin D; PMS: Premenstrual syndrome; OR: Odds ratio; CI: Confidence interval; NHS2: Nurses’ Health Study II; BMI: Body mass index; PMDD: Premenstrual dysphoric disorder; RAAS: Renin-angiotensin-aldosterone system.

## Competing interests

The authors declare no competing interests.

## Authors’ contributions

EBJ, SEH, NGF, SIP, WCW, SRJ, and JEM designed research; EBJ, SEH, WCW, and JEM conducted research; EBJ, SEH, and JEM analyzed data; EBJ drafted manuscript; EBJ, SEH, NGF, SIP, WCW, SRJ, and JEM edited manuscript for important content; EBJ and JEM had primary responsibility for final content. All authors have read and approved the final manuscript.

## Pre-publication history

The pre-publication history for this paper can be accessed here:

http://www.biomedcentral.com/1472-6874/14/56/prepub
